# Impact of Dry Hopping on Beer Flavor Stability

**DOI:** 10.3390/foods10061264

**Published:** 2021-06-02

**Authors:** Bradley M. Titus, Larry A. Lerno, Jordan W. Beaver, Nadia K. Byrnes, Hildegarde Heymann, Anita Oberholster

**Affiliations:** 1 Department of Food Science and Technology, University of California Davis, 1 Shields Avenue, Davis, CA 95616, USA; bmtitus@ucdavis.edu; 2 Department of Viticulture and Enology, University of California Davis, 1 Shields Avenue, Davis, CA 95616, USA; lalerno@ucdavis.edu (L.A.L.); nkbyrnes@ucdavis.edu (N.K.B.); hheymann@ucdavis.edu (H.H.); 3 Department of Chemistry and Biochemistry, University of Texas at Tyler, 3900 University Boulevard, Tyler, TX 75799, USA; jbeaver@uttyler.edu

**Keywords:** beer, dry hopping, flavor, stability, iso-α-acids, polyphenols, sensory

## Abstract

To investigate the chemical and sensorial impact of dry hopping time on typical pale ale, a standardized beer was produced and separated into ten vessels. Nine vessels were dry hopped, and one vessel remained un-hopped as a control. Impact of dry hopping contact time was investigated over 96 h. Polyphenols and iso-α-acid *t*/*c* ratio were analyzed in both Young and Aged beer samples. Total polyphenol content generally increased in both young and aged treatments compared to controls. Analysis of the *t*/*c* ratio suggests that both Young and Aged beers were chemically preserved to some degree after approximately 12 h at the given dry hopping rate regardless of age. Within the Aged beer trials, 96 h of dry hop contact yielded a significant increase in *t*/*c* ratio compared to all other Aged trials. This suggests that a 4-day dry hop regime may yield additional oxidative protection of iso-α-acids in beers stored unrefrigerated for 30 days. Descriptive analysis was also performed with an 8-person, trained panel; however, beers were sensorially distinguished by their aging time as opposed to their dry hopping time.

## 1. Introduction

Hopping can occur at multiple points during beer production, with each point of addition imparting different hop qualities to the beer. The addition of hops during the boil is primarily used to impart bitterness, as the active aroma compounds evaporate out of solution as a function of wort temperature, environmental pressure, and exposure time to the boiling wort (i.e., addition time) [1]. Hopping later during the boil time or after flame-out often imparts more aromatic qualities of the hops, as the lower temperatures retain the volatile oils in solution more effectively [2] (pp. 133–135). “Dry hopping” is a method of potentially imparting even higher concentrations of aroma into the finished beer without contributing significantly to bitter taste. This occurs when hops are added post-fermentation, allowing aroma compounds to slowly extract into the alcoholic solution while minimizing extraction of bittering acids from the solid material due to the lack of high heat [3]. While modern dry hopping is primarily used to impart beers with the characteristic hoppy aroma, earlier work from the eighteenth century describe dry hopping as a method preserving the freshness and flavor of beer, as well as limiting microbial growth [4]. The stability of beer flavor, as well as how beer chemistry contributes to flavor stability, has long been a subject of interest to researchers. As many of the hop-derived compounds-such as polyphenols, humulones, and iso-α-acids-act as antioxidants and demonstrate antimicrobial properties, it can be expected that they will have some effect on flavor stability and the aging potential of beer. While the exact nature of beer flavor stability is not fully understood, it is clear that aldehydes and other byproducts of oxidation play a major role [5,6,7].

Depending on the beer style, hops may contribute up to 50% of the total polyphenols in beer, with the remainder being primarily derived from barley [8]. While the extraction of polyphenols occurs throughout the brewing process, the greatest rate of extraction in dry hopped beers occurs in the first twelve hours, where approximately 80–90% of the hop-derived polyphenols extract [9]. The classes of polyphenols that extract into beer are hydroxybenzoic acids, hydroxycinnamic acids, flavan-3-ols, and flavonols (including their glycosides), proanthocyanidins, prenylchalcones, and stilbenes [10,11,12]. These polyphenols contribute significantly to the quality of the finished product in many ways. All of these classes of compounds display antioxidant properties, with the flavan-3-ols and flavonols showing the greatest radical scavenging abilities [13]. The polyphenols also contribute to taste and mouthfeel, especially the flavan-3-ols and proanthocyanidins, which are bitter and responsible for the sensation of astringency. The flavan-3-ols and proanthocyanidins may potentially play roles in both colloidal and foam stability, though further data is needed to substantiate this claim [14]. The wide range of concentrations at which polyphenols are found in beer combined with the complex matrix makes the analysis of beer polyphenols difficult. Methods have been published utilizing solid phase extraction (SPE) for the isolation and concentration of polyphenols followed by reversed-phase HPLC (RP-HPLC) for identification and quantification [15]. A major limitation of these methods is low recoveries for several polyphenols. An alternative analytical platform that is increasingly being used is RP-HPLC coupled to mass spectrometry. A 2015 study by Quifer-Rada, et al. showed successful implementation of a high-throughput HPLC-OrbitrapMS method to identify and quantify 47 distinct phenolics in four different styles of beer [10].

A second class of hop-derived compounds are the iso-α-acids (IAA), which are the primary contributors to bitterness in beer [2] (p. 127). In the hop, these compounds are found primarily as α-acids with small amounts of β-acids (about 3% by weight) [16]. The former of these bittering acids is isomerized to “iso-α-acids” during the boil which increases its solubility in aqueous matrices as well as intensifies its bitterness perception [17]. Six IAA are found in beer, these being *cis* and *trans* isomers of iso-humulone, iso-cohumulone, and iso-adhumulone. While both *cis* and *trans* IAA are bitter, the *cis* isomer has been shown to be more bitter as well as being slightly more thermodynamically stable, degrading at a slower rate than the *trans* isomer. The primary degradation pathways for IAA are acid-catalyzed degradation, oxidation, and photooxidation. The difference in degradation rates has been used to develop a metric of beer flavor stability, the *trans*-to-*cis* ratio (*t*/*c*), which is the ratio of the sum of the *trans* isomers to that of the sum of the *cis* isomers [18]. For most freshly brewed conventionally hopped ales the IAA content is approximately 30% *trans* IAA and 70% *cis* IAA. It has been suggested that the *t*/*c* ratio correlates with stale flavor intensity as qualified organoleptically by trained panelists and can be used as a marker of beer quality [19,20].

The compounds responsible for aroma and bitterness from hops are located in the lupulin glands [2] (pp. 127–128). Efficient extraction of these compounds relies on the lupulin glands being ruptured, most often by chemical or physical means. To allow for efficient extraction, most commercial hops are processed into pellets that breakdown the structure of the flower [17]. Chemical breakdown can occur by thermal degradation (such as during the boil) or solvent and hydration effects. As dry hopping occurs at reduced temperatures, the primary means of chemical breakdown would be from exposure to the hydroalcoholic environment of the finished beer. Surprisingly, few studies have investigated how hopping duration will affect the extraction of hop-derived compounds and concurrently how hopping duration will affect beer flavor stability. One of the only controlled studies to investigate this seemingly important production process is by Lafontaine and Shellhammer [21]. In pilot-scale fermentors, a standardized pale ale was dry hopped at four different concentrations for 24 h. Additionally, an un-hopped control batch was simultaneously produced. The results suggested that the relationship between dry hopping rate and both sensorial and chemical profiles of the finished beer was non-linear. The authors suggested that optimal dry hop concentration is between 400 and 800 g hops/hL beer, a wide range. However, this study only investigated the dry hopping process over 24 h, whereas most industry productions allow hops to remain in contact with beer for much longer periods. As it stands, the current body of knowledge is lacking with regard to this production process.

To determine how dry hopping time affects beer flavor stability, a standard ale was brewed and split into lots which were then hopped with increasing exposure times. The finished beers were analyzed by RP-HPLC to determine both polyphenolic content and *t*/*c* ratio of iso-α-acids. Additionally, descriptive analysis was performed to determine sensory impact. All treatments were reanalyzed after 30 days at room temperature in order to determine how the different hopping treatments affected the aging potential of the beers.

## 2. Materials and Methods

### 2.1. Reagents

Protocatechuic acid, 4-hydroxybenzoic acid, gallic acid, vanillic acid, syringic acid, *p*-coumaric acid, sinapic acid, chlorogenic acid, caffeic acid, ferulic acid, (+)-catechin, (-)-epicatechin, (+)-catechin gallate, (-)-epicatechin gallate, quercetin, quercetin-3-glucoside, *p*-fluorobenzaldehyde, and *O*-(2,3,4,5,6-pentafluorobenzyl)-hydroxylamine (PFBHA) were purchased from Sigma Aldrich (St. Louis, MO, USA). Acetonitrile (HPLC grade), methanol (HPLC grade), *ortho*-phosphoric acid (85%, HPLC grade), glacial acetic acid (HPLC grade), and sodium chloride (ACS reagent grade) were purchased from Thermo Fisher Scientific (Waltham, MA, USA). Water purified to a final resistance of 18 MΩ using a Milli-Q filtration system (Millipore Sigma, Burlington, MA, USA) was used. Dicyclohexylamine salts of *trans*-iso-α-acids (ICS-13, 62.3% *w*/*w*) were from the American Society of Brewing Chemists (St. Paul, MN, USA).

### 2.2. Beer Production and Dry Hopping

All beer used in this study was brewed at the August A. Busch III Brewing & Food Science Laboratory (pilot brewery) at the University of California, Davis (Davis, CA, USA). Two 1.7 hL batches were brewed consisting of 94.2% Pale Ale Malt (Rahr Malting Co., Shakopee, MN, USA) and 5.8% Caramalt 15 L (Crisp Maltings, Norfolk, UK). A total of 200 mg/L Ca^2+^ was added using CaCl_2_ and CaSO_4_ in a 1:1 ratio into the mash and the pH was adjusted to 5.3 for both batches with 88% (*w*/*v*) lactic acid solution (BSG Handcraft supplies, San Leandro, CA, USA). The mash was conducted in a steam-jacketed mash vessel with an agitator for mixing and a ramp rate of 1 °C/min. The strike water was 65 °C and the 3 L per Kg of malt resulted in an initial mash temperature of 59 °C. The mash was immediately ramped to 65 °C and held for 60 min for conversion before heating to a mash out temperature of 77 °C. The mash was held at mash out for 5 min before transferring to the lauter and sparging with 77 °C deionized water to reach a kettle full volume of 1.8 hL. An addition of Magnum hops (15.2% α-acids, BSG Handcraft supplies, San Leandro, CA, USA) was added at the beginning of the 60-min boil to achieve 30 IBUs. Refined carrageenan (20 ppm, Murphy and Son Limited, Nottingham, UK) and yeast nutrients (5 g/hL, Lallemand, Milwaukee, WI, USA) were added at the end of the boil. At flame-out, the original gravity of the wort was 12.0 ^o^P. After the whirlpool the wort was sent to fermenters at a rate of 7.57 L/min and injected in-line with 0.12 L/min oxygen for a prefermentation oxygen concentration of 12 mg/L. Glycol jacketed fermentors were set to 18 °C before inoculation with American Ale Yeast–Wyeast 1056 (Wyeast Laboratories, Inc., Hood River, OR, USA) at a rate of 1.0 × 10^6^ cells/mL/^o^P. Beers were fermented to a terminal gravity of 2.6 ^o^P in 72 h. After 168 h and a diacetyl rest, the yeast was dumped, and the beer was cold crashed to 0 °C for seven days. The beer was then blended and filtered to remove yeast prior to hop treatment. The blended beer was transferred to 11 L vessels and dry hopped in triplicate at a rate of 8 oz/bbl (1.43 g/L) with Cascade hops. The control beer was also transferred to 11 L vessels and was not dry hopped. Hop pellets (T-90, BSG Handcraft supplies, San Leandro, CA, USA) were introduced to each treatment for a given contact time. Contact times investigated in this study were: 1 h, 2 h, 3 h, 6 h, 12 h, 24 h, 48 h, 72 h, and 96 h. Treatments were sampled into 2 L PET bottles attached with stainless carbonation caps allowing the bottles to pressurized and purged with CO_2_ prior to filling and were carbonated with 2.5 volumes of CO_2_ prior to sensory or storage. “Aged” beer samples were stored at room temperature for 30 days to represent the recommended shelf stability for beer in commercial retail stores.

### 2.3. Iso-α-Acids

#### 2.3.1. Isolation of Iso-α-Acids

Iso-α-acids (IAA) were isolated from samples by solid phase extraction (SPE) using a method based on that of Donley [22]. All SPE was performed using Bond Elut C8 columns packed with 500 mg of stationary phase (Agilent Technologies, Santa Clara, CA, USA). SPE columns were conditioned with 2 mL of methanol followed by 2 mL of water. Beer samples (25 mL) were acidified to pH 2.8 with *O*-phosphoric acid and loaded onto the SPE column and washed with 6 mL of water:phosphoric acid (99.8:0.2) followed by 2 mL of methanol:water:phosphoric acid (49.9:49.9:0.2). IAAs were eluted with 5 mL of methanol:phosphoric acid (99.9:0.1) into 5 mL volumetric flasks and the final volume was adjusted to account for solvent remaining in the SPE column. Eluent was filtered through a 0.2 µm filter prior to analysis by HPLC.

#### 2.3.2. RP-HPLC of Iso-α-Acids & Calculation of Trans/Cis Ratios

Isolated IAAs were analyzed by RP-HPLC using a method based on Jaskula et al. [23]. All analyses were performed on an Agilent 1260 Infinity liquid chromatograph (Agilent Technologies, Santa Clara, CA, USA) equipped with a temperature controlled autosampler, binary pump, thermostatted column compartment, and a diode array detector. Instrument control and data analysis was performed in Agilent (Santa Clara, CA, USA) Chemstation (version D.04). Isocratic separations were performed on an Alltech (Columbia, MD, USA) Altima column (150 mm × 4.6 mm). Eluent A consisted of 18.2 MΩ deionized water adjusted to a pH of 2.80 with phosphoric acid. Eluent B was acetonitrile. The mobile phase consisted of 48% solvent A and 52% solvent B with a flowrate of 1.8 mL/min. Eluting peaks were monitored at 270 nm and 314 nm and were compared to the IAA standards from the American Society of Brewing Chemists for identification. The ratio of *trans* to *cis* IAAs was calculated for each chromatogram using integrated peak areas and Equation (1).

(1) $$\frac{T}{C}=\frac{\left(t-isocohumulone\right)+\left(t-isohumulone\right)}{\left(c-isocohumulone\right)+\left(c-isohumulone\right)}$$

### 2.4. Polyphenols

#### 2.4.1. Sample Preparation and Standard Addition

Analytical standards (95–98% purity) of 4-hydroxybenzoic acid, protocatechuic acid, gallic acid, vanillic acid, syringic acid, p-coumaric acid, sinapic acid, chlorogenic acid, caffeic acid, ferulic acid, catechin, epicatechin, catechin gallate, epicatechin gallate, quercetin, and quercetin-3-glucoside were obtained (Sigma Aldrich, Milwaukee, WI, USA). A mixture of the polyphenols of interest was prepared in methanol from chemical standards and stored under nitrogen at −20 °C. The concentration of each standard was chosen so that subsequent spikes resulted in concentrations previously reported in related literature [11,12]. Beer samples were centrifuged, four aliquots of 1 mL were taken and three were spiked with increasing volumes of the polyphenol mixture. Spiked and non-spiked aliquots were centrifuged at 13,200 rpm for five minutes and the supernatant was transferred to HPLC vials prior to analysis.

#### 2.4.2. LC/MS/MS Analysis of Polyphenols

Samples were analyzed on an Agilent (Santa Clara, CA, USA) 1260 Infinity HPLC connected to an Agilent 6430 triple quadrupole mass spectrometer equipped with an electrospray ionization source. Chromatographic separations were performed on an Alltech Altima (Grace, Deerfield, IN, USA) column (4.6 mm × 150 mm, 3 μm) using a binary solvent system of water acidified with 1% glacial acetic acid (Solvent A) and acetonitrile acidified with 1% glacial acetic acid (Solvent B). The gradient used for separation was as follows: 3% B at 0 min, 25% B at 50 min, 80% B at 55–60 min and 3% B at 65–75 min. The flow rate during analysis was 1.25 mL/min and the flow was split via a post-column tee so that the flow rate to the electrospray source was approximately 0.3 mL/min. An injection volume of 1.5 μL were used for all analyses. The electrospray ionization source conditions were drying gas temperature of 350 °C, drying gas flow 13 L/min, nebulizer pressure 30 psi, and capillary voltage 4000 V for positive mode and 2500 V for negative mode. The monitored transitions for each phenolic compound are shown in Appendix A Table A1 and *R*^2^ values for injection volumes are displayed in Appendix A Table A2. Instrument control and data analysis was performed using the Agilent (Santa Clara, CA, USA) MassHunter Acquisition and Qualitative Analysis software respectively (both version B.04). The concentration of polyphenols in the unspiked sample was determined by fitting a linear regression to the plot of the integrated peak area versus spiked concentration. All fits were highly linear with all *R*^2^ greater than 0.99.

### 2.5. Descriptive Analysis

#### 2.5.1. Panelists and Ballot Development

The descriptive analysis portion of this study received approval from the University of California, Davis Institutional Review Board (IRB 676977-1). All participants gave informed consent to participate in the study. All experimental beer treatments (*n* = 30) were assessed by eight trained panelists (six male and two female) from the student body of the University of California, Davis and all proclaimed to be regular craft beer drinkers. Each panelist attended training sessions over two weeks to familiarize them with the characteristics of dry hopped beer and build the lexicon of attributes against which the beers would be evaluated. Ballot training was used in this study and panelists were presented with an initial ballot of terms commonly used to describe beers. During training, panelists modified the ballot based on sensory characteristics of the beers in the study, coming to a consensus on a final ballot of thirteen attributes (Table 1).

#### 2.5.2. Evaluation of Experimental Beers

Prior to each evaluation the panelists were given a quiz designed to calibrate them against the thirteen attributes. Reference standards were presented in evaluation booths in black glasses with random blind-labeled three-digit codes. Panelists had to correctly identify all thirteen standards before proceeding to the evaluation of the experimental beers. If any standard was incorrectly identified the panelist was asked to smell the provided labeled standards again.

Ten beers were presented to each panelist in an isolated booth during each evaluation. Beers were served in clear, straight tasting glasses with lids (glass volume was 148 mL) labeled with a three-digit blinding code. All samples were presented in a Latin square design over the course of the experiment. To combat fatigue and desensitization, the ten beers were evaluated in two sets of five with a ten-minute break between. Between each beer, the panelists were instructed to sip water and wait one minute to cleanse their palate. Evaluations were done by removing the lid after swirling the glass to create foam and release aromatic compounds. The panelists were instructed to begin with one short sniff followed by one long sniff of the beer. After this they were to sip the beer, swish the beer around their mouth, and expectorate. Panelists were allowed to repeat this procedure if necessary. Each panelist evaluated the beers against each of the thirteen attributes on a line scale. Data was recorded in the sensory analysis software FIZZ (Biosystems, Burgundy, France). The different beer treatments were evaluated twice. The first evaluation occurred immediately after the dry hop treatment and the second evaluation occurred after 30 days of aging at room temperature. Evaluation procedures were identical during both evaluations and each evaluation took place over two weeks.

### 2.6. Data Analysis

All data was analyzed using JMP (SAS Institute, Cary, NC, USA) and R-Studio (The R Foundation, Affero General Public License). For *t*/*c* ratio data, analysis of variance (ANOVA) and Tukey HSD post hoc tests were performed to test for statistical differences among treatments. For polyphenols, Student’s *t*-tests were performed to determine significance between young and aged samples within each timepoint (α = 0.05). Sensory data was analyzed using canonical variate analysis (CVA) with Wilk’s Lambda to test against the null hypothesis (α = 0.05). Bartlett’s test was used for eigenvalue significance. Multifactor analysis (MFA) was performed to determine correlations among treatments considering all chemical and sensorial data collected.

## 3. Results

### 3.1. Iso-α-Acids

**Figure 1:**
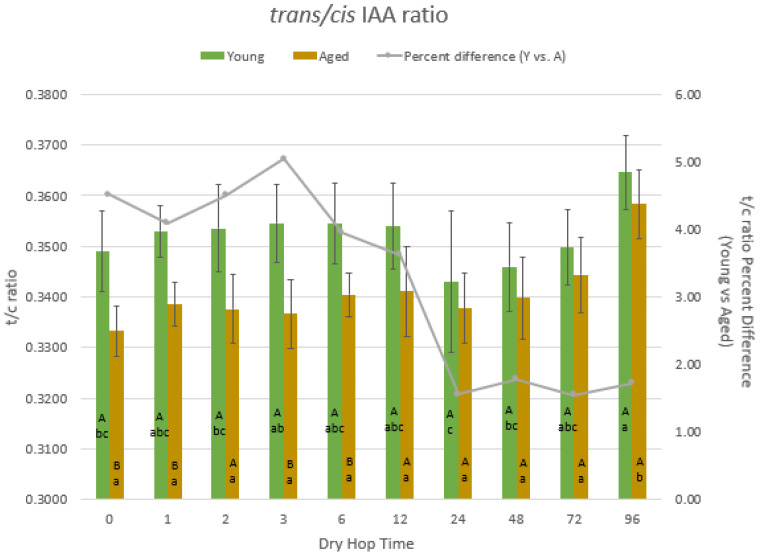
Comparison of the *t*/*c* ratios for each pair of Young and Aged treatments and the %*t*/*c* ratio loss during aging (*n* = 9). Upper case letters = significance difference between Young and Aged beers with the same dry hop time. Lower case letters = significance difference between dry hop time within the same storage treatment.

Iso-α-acids were isolated from fresh and aged beers samples by SPE and analyzed by RP-HPLC. The average *t*/*c* ratios were calculated and compared as a function of both storage of beer (Young vs. Age with same dry hopping time) and dry hopping time (within the same storage treatment). Shown in Figure 1, the average *t*/*c* ratio of the Young beers ranged from 0.343 to 0.365, which are typical of fresh beers and similar to values reported in literature [20,23]. After 6 h of dry hopping time, there was no significant difference in *t*/*c* ratio (oxidative protection) between Young and Aged beers. However, within the Aged trial, the 96-h trial was statistically distinguishable from all other time points. Within the Young treatment, the 96-h treatment had the largest average *t*/*c* ratio, but it was not statistically distinguishable from the 1–12 or 72-h dry hopping trials. This statistical overlap is potentially due to variability within the IAA measurements or due to IAAs in younger beer being more protected from oxidation by malt-derived antioxidants that had dissipated within the Aged beer over storage time. However, the Young 96-h measurement is still significantly higher than the 24- and 48-h trials, potentially suggesting that, regardless of the a beer’s age, a longer dry hopping time may aid in the overall protection of IAAs.

During the aging process, the *t*/*c* ratio decreased an average of 1–5% by comparison to Young beer, with hopping treatments less than 24 h having the greatest change. For the 0–6 h treatments, the differences in *t*/*c* ratio between Young and Aged beers were mostly significant, while the 12–96 h treatments were statistically similar. This strongly suggests that after approximately 24 h of dry hopping at the rates used in this experiment (1.43 g pellets per liter of beer), the beers were chemically preserved to some degree. As the *t*/*c* ratio reflects the degree of oxidative degradation of the beer, it was hypothesized that the increased hopping time allowed for greater extraction of polyphenolics, the primary antioxidants in beer [8].

### 3.2. Phenolic Compounds

The phenolic content of the hopping treatments was determined by RP-HPLC-MS/MS for both the young and aged beers. The phenolics monitored were those that have been shown in literature to be in the greatest abundance in beer [12]. The phenolics monitored were gallic acid, protocatechuic acid, p-hydroxybenzoic acid, vanillic acid, chlorogenic acid, sinapic acid, caffeic acid, p-coumaric acid, ferulic acid, (+)-catechin, (-)-epicatechin, (+)-catechin gallate, (-)-epicatechin gallate, quercetin-glucoside, and quercetin. During initial method development utilizing a pooled sample of the fresh beers it was determined that (+)-catechin gallate and (-)-epicatechin gallate were not detectable in the beers made for this study and were therefore not monitored, even though they are shown as being abundant in literature. The use of tandem mass spectrometry for the identification and quantitation of the phenolics was necessary as the phenolic content in the beers was below the limit of detection of traditional diode array detectors (DAD) used in RP-HPLC methods. Attempts to isolate and concentrate phenolics by SPE were not successful, with recoveries of less than 50% being observed for the majority of the monitored phenolics (data not shown). Quantitation of the monitored polyphenols was performed by standard addition, using three concentration spikes per phenolic compound.

**Figure 2:**
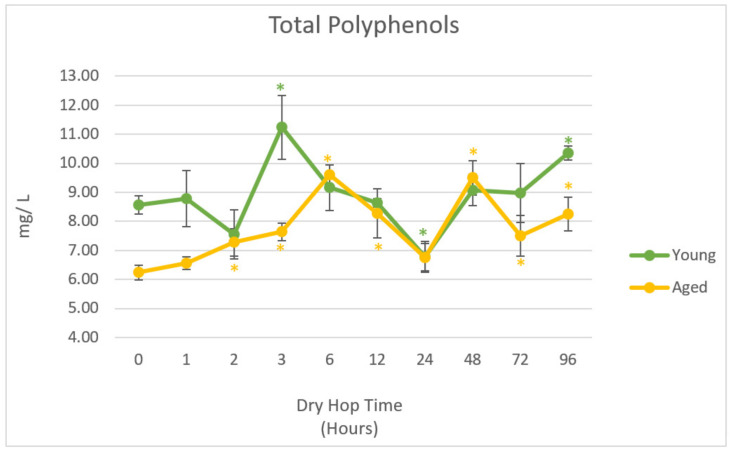
Summation of polyphenol concentrations (mg/L) measured in Young and Aged beer (*n* = 3). Statistical significance compared to control beers (*t* = 0) only within the same aging treatment. *: α = 0.05.

The measured concentration of individual polyphenols in the Young and Aged beer are shown in Appendix A Table A3. The summed concentrations of all polyphenols over time for Young and Aged treatments are shown in Figure 2. Statistical significance of dry hopped trials was only compared with regard to control treatments within each storage treatment. Generally, the greatest concentration of phenolics were in the 3- and 96-h hopping treatments of the Young beer (11.16 and 10.29 mg/L total phenols respectively). Within the Aged treatment, the majority of timepoints show a significant increase in polyphenol content by comparison to control beer after 2 h of dry hop contact.

Within the Young treatment, there were only two timepoints which showed significant increases in total phenolics compared to non-dry hopped beer: the 3-h and 96-h timepoints. In terms of kinetics, these results are somewhat confounding as polyphenol concentration markedly decreased lower than the control concentration after 24 h followed by an increase in concentration within the last 3 days of dry hop exposure. The relative decrease in polyphenol content in the longer hopping time treatments may be due to oxidative losses during storage and may explain the similar trends observed for the iso-α-acids *t*/*c* ratios (Figure 1). Alternatively, the data may potentially display a shifting equilibrium of polyphenol extraction and re-adsorption from the solid hop particles. This hypothesis is further discussed in Section 4.

In general, the trend in concentration of polyphenols in the Aged beer was similar to that of the Young beers, although the greatest average concentration was in the 6-h treatment (9.60 mg/L), and no dry hop trial showed polyphenol concentration statistically below that of the Aged control. The concentration of several polyphenols decreased with aging, most notably (+)-catechin and (-)-epicatechin (Table A3).

**Figure 3:**
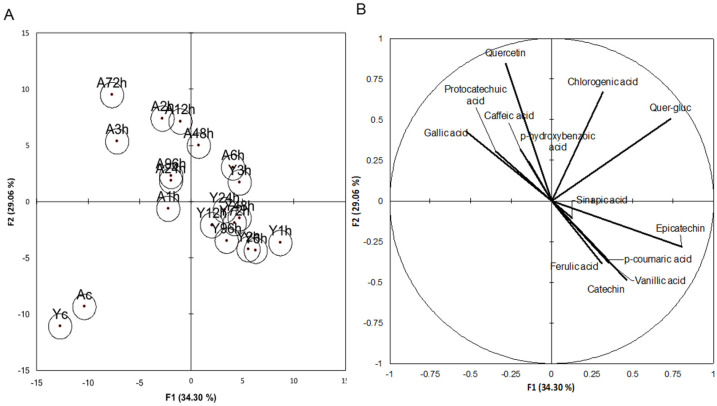
(**A**) Canonical Variance Analysis of young (Y) and aged (A) beers. “XXh” = hours, “c” = control (*n* = 3). (**B**) Variable plot of measured polyphenolics.

Discriminant analysis was additionally performed in order to determine the primary differences among the treatments using classifications of Young and Aged beer and specific polyphenols (Figure 3). The results of this analysis showed separation among the control beers, the Young beers, and the Aged beers. The Young and Aged control beers were similar to each other and were well separated from either the Young or Aged hopping treatments. With regards to the hopping treatments, no clear trend was discerned based on hopping time; rather, the separation was by storage time. Furthermore, the Young beers had fewer differences among the treatments than the aged beers, evidenced by the spread in centroids.

The variables plot (Figure 3B), showed that the Young beers were characterized by sinapic acid, p-coumaric acid, ferulic acid, (+)-catechin, and (-)-epicatechin while the Aged beers were characterized by quercetin, gallic acid, protocatechuic acid, p-hydroxybenzoic acid, and caffeic acid. The implication of the discriminant analysis results is that any amount of hopping led to differences in aging potential for the beers made for this study.

### 3.3. Descriptive Analysis

**Figure 4:**
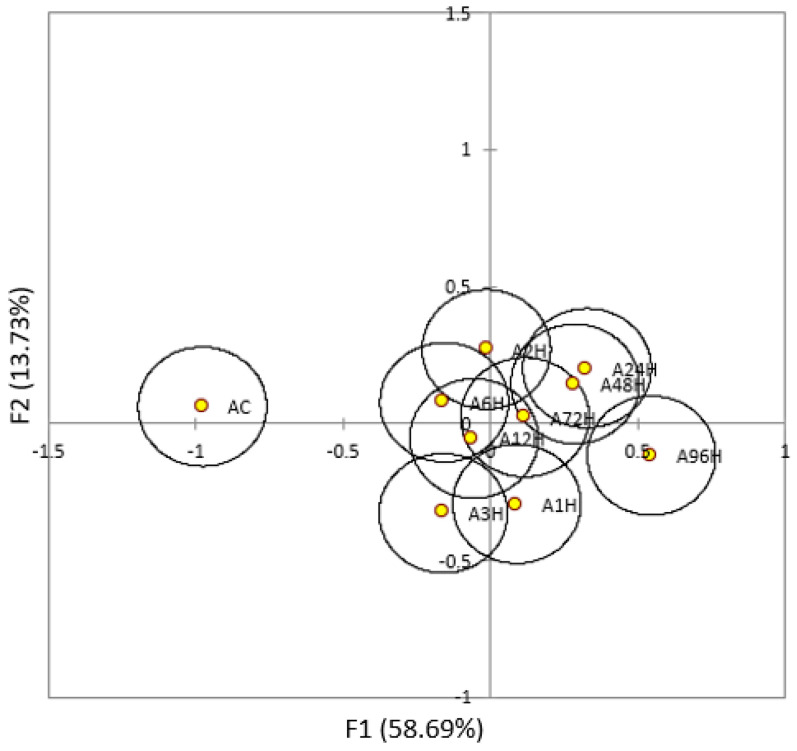
CVA score plot of the aged treatments showing 95% confidence intervals (*n* = 3).

**Figure A1:**
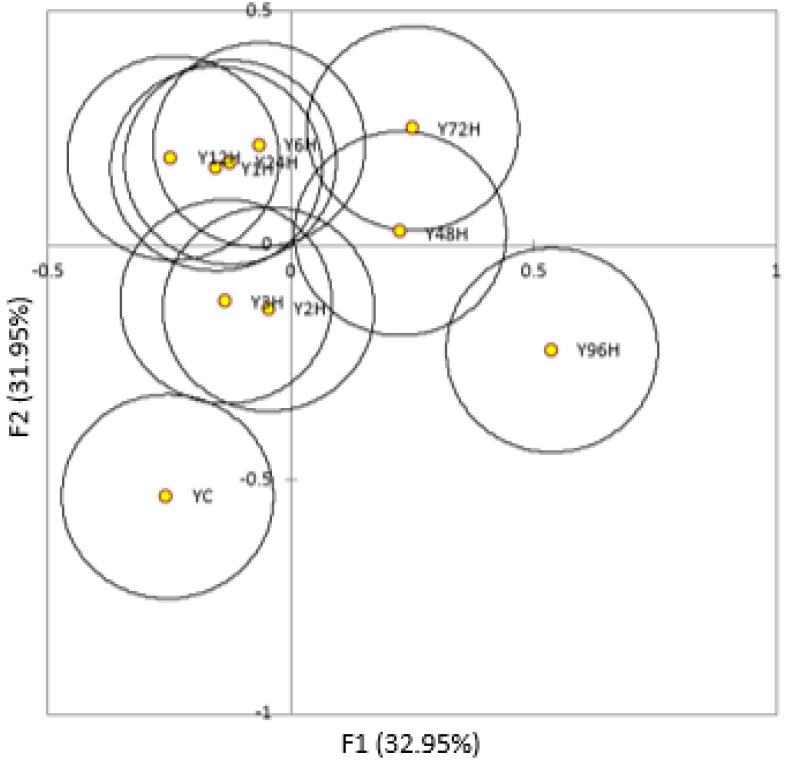
Canonical variates analysis (CVA) score plot of young treatments showing 95% confidence intervals and groupings.

Young and Aged beers were analyzed by descriptive analysis to determine if the hopping treatments had an effect on beer flavor quality and stability. A panel of trained judges generated a list of thirteen attributes that described the young beers in the study (Table 1). The same list of attributes was utilized for the analysis of the Aged beers. Descriptive analysis of the Young beers showed that panelists were not able to differentiate among the hopping treatments (Wilk’s Lambda test failed to reject null hypothesis at (α = 0.05)), but passion fruit, stone fruit, pineapple, grapefruit, citrus, and vegetable attributes were distinguishable between Young and Aged beers. It should be noted that the Young control sample separated from the other dry hopped Young beers on the CVA plot (Appendix A Figure A1), though not enough to be considered statistically different. Comparing within the Aged treatments, the control was statistically different from all dry hopped trials (Figure 4). These results suggest that, over time, a beer that has not been dry hopped will age differently than beers that were dry hopped.

**Figure 5:**
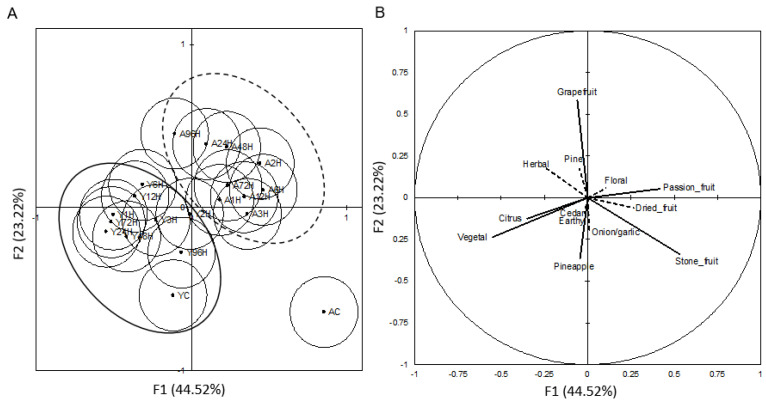
Canonical variance analysis of the sensory data for the young (Y) and aged (A) beers with different hop exposure times. (**A**) Score plot showing the grouping of young (solid oval) and aged beers (broken line oval) (*n* = 3). (**B**) Variable plot showing the ballot attributes that are significant (solid line) and not significant (broken line). Confidence intervals were determined at 95%.

As both the Young and Aged beers clustered together (showing few statistical differences), the descriptive analysis results for the fresh and aged beers were compared by CVA to determine if the Young and Aged beers could be differentiated from each other based on sensory characteristics. The CVA of the combined sensory data for the fresh and aged beers is shown in Figure 5. Separation of the fresh and aged beers occurred along both factors 1 and 2. A slight trend was observable, with the extreme hopping treatments (control and 96-h) being more removed from the intermediate hopping times. Interestingly, the Aged control beer was well separated from the other aged treatments while the fresh control treatment was only slightly separated. Again, these results suggest that over a longer storage time a beer that has not been dry hopped will age differently than beers that have, supporting previous observations that any hop contact resulted in increased aging potential and flavor stability. This mirrors the results of the polyphenols analysis, where the presence or absence of hops was responsible for the separation of treatments (Figure 3).

**Figure 6:**
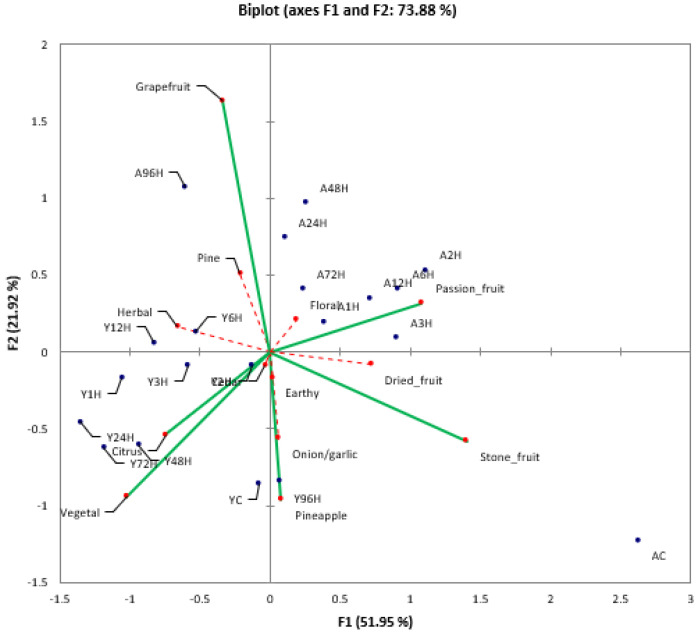
CVA loadings plot of the descriptive analysis ballot attributes and sample treatments. Y = Young, A = Aged (*n* = 3).

As previously mentioned, the attributes found to be different between the Young and Aged beers were passion fruit, stone fruit, pineapple, grapefruit, citrus, and vegetable (Figure 6). The loadings plot shows that the aging process appears to be driven by stone fruit, passion fruit, and grapefruit. The attribute most highly associated with the control Aged beer was stone fruit. It has been shown that many lactones [24,25,26,27], aldehydes [27] and thiols [28] formed from oxidation reactions are associated with a similar aroma descriptions. Though these aroma compounds were not successfully measured within this experiment, it stands to reason that the control beers would be more highly associated with these aroma descriptors. Most of the dry hopped trials, particularly within the Aged treatment, showed significantly higher concentrations of polyphenols and *t*/*c* ratios by comparison to control beer. This increase suggests an aid in oxidative protection of the dry hopped beers, potentially reducing the formation of the aforementioned stone fruit aroma compounds, particularly during the extended, room temperature storage of the Aged beers. Alternatively, hop-derived compounds may have simply masked the perception of oxidation products. Mono- and sesquiterpenes found in hops have been shown to be highly associated with floral and citrus aromas [29]. As shown in Figure 5, dry hopped treatments generally trend more towards those descriptors by comparison to the control beers, regardless of age. Chemical validation is needed to substantiate these claims within this experiment, but the origin and impact of these compounds within the brewing process is well documented [30].

## 4. Discussion

Unsurprisingly, results of this investigation suggest that dry hopping has preservative effects on beer flavor stability. Beers dry hopped for at least 24 h generally showed less IAA degradation during prolonged, room-temperature storage than beers that were dry hopped for shorter times based on the iso-α-acids *t*/*c* ratio–a known chemical aging marker in beer. The descriptive analysis studies showed that dry hopping for periods as short as one hour was enough to organoleptically differentiate the beers from the control treatment after aging. This is evidence of rapid extraction of hop-derived compounds during dry hopping at room temperature. However, beer aging seemed to have a larger impact on the sensory attributes of the beers than dry hopping time. Perhaps the impact of dry hopping would have been more apparent with the use of a more aggressive dry hopping regime or a longer dry hop contact time. Additionally, no stale beer characters were rated within the descriptive analysis due to the fact that the same attribute list was scored for both Young and Aged beers. This was ultimately due to time constraints in training a panel in new attributes and evaluating the beers within the targeted aging period. Future sensory studies containing stale beer characters could potentially separate beers more clearly according to known aging characters.

The HPLC analysis of the iso-α-acids *t*/*c* ratio and phenolics were significantly more reproducible. Sufficient chromatographic separation and reproducibility was achieved through EDTA addition to solvent A. Interestingly, the polyphenol content decreased between 3 and 96 h, rather than the hypothesized increase with hopping time, with the total phenolic content in the 24-h treatment being approximately 24% less than the 3-h treatment. This decrease in phenolic content is contrary to expectations, as one would expect increased extraction with increasing contact time with hop pellets. This decrease in concentration may be indicative of several processes, including precipitation of extracted polyphenols, adsorption to solids (such as yeast hulls or hop cell wall material), or oxidative degradation [14,31]. One potential hypothesis of the fluctuating polyphenol trend is that pelletized hops initially introduced into solution expectedly increased total polyphenols in solution in the first 3–6 h of dry hop contact with beer. However, as pellets began to absorb liquid and break apart, their surface area significantly increased. Following assumptions from Langmuir model liquid-solid adsorption systems, this increase in surface area of adsorbent solid material in the fermentor may have shifted the extraction equilibrium of the system to selectively adsorbed polyphenols from solution [32]. Then, over the 48–96-h window, polyphenols desorbed from the solids, as hop cell wall material was gradually hydrolyzed or solubilized-decreasing the adsorbent surface area. This has been shown in wine-like systems [31,33], but a thorough investigation into the fates of extracted polyphenols during the brewing process is needed to fully understand what was observed in this study.

**Figure 7:**
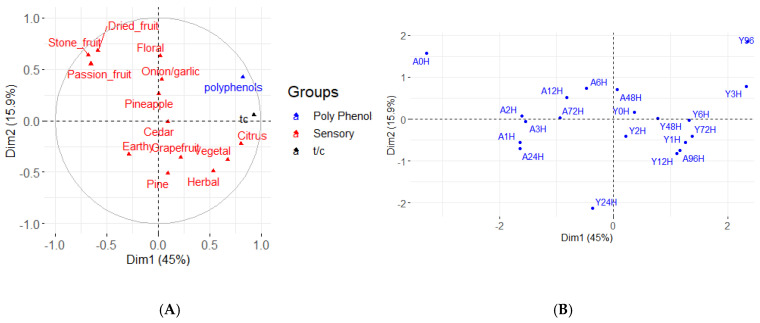
Multifactor analysis of *t*/*c* ratio, polyphenols, and sensory data of Young (Y) and Aged (A) beers. (**A**). Variable plot. (**B**) Score plot of individual treatments.

Multifactor analysis (MFA) of the *t*/*c* ratios, total polyphenols, and descriptive analysis data was performed to determine if any significant correlations existed (Figure 7). However, the only noticeable trend was similar to the sensory data in that separation of the treatments was mostly a function of storage time. It is worth noting that there was clear separation of the Aged control with all other dry hopped Aged beers, and that the 96-h treatments of both Young and Aged beers trended towards citrus fruit aroma, higher *t*/*c* ratio, and higher polyphenol concentrations. However, there was not a noticeable trend with regard the kinetics of dry hopping time, and the first two dimensions of the MFA only represent 60.9% of the variance. The third dimension (13.3%) was analyzed, but no other noticeable correlations were seen.

Ultimately, the data from this experiment suggests that the process of dry hopping chemically distinguished both Young and Aged samples from control treatments, but Aged beers were more significantly impacted by the factor of dry hop time. Future research utilizing small-scale, model beer solutions may reduce the complexity of the matrix and procedural variably enough to elucidate time-dependent trends. Long term studies on the impact of hop-derived polyphenols during dry hopping as well as investigation into the extraction of oligomeric and polymeric polyphenols could benefit the current knowledge base. Condensed tannins extracted from hops affect beer flavor and colloidal stability, and their quantification during beer aging will help obtain a better understanding of phenolic evolution in beer.

## Figures and Tables

**Table 1:** Descriptive analysis aroma references and attributes.

Attribute	References Standard
Passion fruit	Fresh pulp of ¼ of a fresh passion fruit
Onion/garlic	Three 1 cm^2^ pieces of raw yellow onion and 20.0 g of raw, chopped garlic
Stone fruit	Four 0.5 cm^3^ pieces of white peach and four 0.5 cm^3^ pieces of nectarine
Pineapple	Six 0.5 cm^3^ pieces of fresh cut pineapple
Pine	One pinch of damp pine shaving with three drops of PineSol (Chlorox, Oakland, CA, USA)
Cedar	One 5 cm × 2 cm piece of cedar shaving soaked overnight in grain alcohol
Grapefruit	Six 0.5 cm^3^ pieces of fresh grapefruit rind
Herbal	Contents of ½ tea bag of green tea (Celestial Seasonings, Boulder, CA, USA) and ½ tea bag of chamomile tea (Stash Tea Co., Tigard, OR, USA)
Floral	Small sprig of fresh picked lavender
Citrus	Six 0.5 cm^3^ pieces of fresh naval orange rind and six 0.5 cm^3^ pieces of fresh lemon rind
Earthy	Two 0.25 cm^2^ pieces of dried Porcini mushrooms (Grapevine Trading Co., Santa Rosa, CA, USA)
Vegetal	One 1 cm^3^ pieces of fresh cut bell pepper, two 2.5 cm frozen green bean pieces, and two frozen oeas (Norpac Foods Inc., Brooks, OR, USA)
Dried fruit	Eight 1 cm^3^ pieces of dried apricot (Sun-Maid, Fresno, CA, USA)

## Data Availability

Sensory data available upon request due to restrictions of panelist privacy. Other data available in ProQuest Dissertation and Thesis Global database (I.D. 1796034783). https://about.proquest.com/en/products-services/pqdtglobal/, accessed on 23 May 2021.

## Appendix A

**Table A1:** Mass spectrometer method energy and m/z values for measured phenolics.

Compound	Elution Order	Time Segment	Fragmentor Value	Collision Energy	Precursor Ion	Product Ion
Gallic acid	1	1	80	12	169	125
Protocatechuic acid	2	2	80	15	153	109
p-hydroxybenzoic acid	3	3	80	15	137	93
Catechin	4	4	130	9	289	245
Chlorogenic acid	5	5	95	12	353	191
Vanillic acid	6	5	80	9	167	123
Caffeic acid	7	6	95	15	179	135
Syringic acid	8	7	80	9	197	153
Epicatechin	9	8	130	9	289	245
p-coumaric acid	10	9	80	15	163	119
Ferulic acid	11	10	80	15	193	134
Sinapic acid	12	11	95	6	223	208
Cat gallate	13	11	115	9	441	289
Epi gallate	14	12	115	9	441	289
Quer-gluc	15	13	130	21	463	301
Quercetin	16	14	115	18	301	151

**Table A2:** Linear regression study for injection volumes for LC-MS/MS method for measured phenolics.

Compound	*R* ^2^			
	1 µL Injection	2.5 µL Injection	5 µL Injection	10 µL Injection
Gallic acid	0.9939	0.9970	0.8519	0.8831
Protocatechuic acid	0.9987	0.9959	0.8378	0.8699
p-hydroxybenzoic acid	0.9998	0.9849	0.8061	0.8406
Catechin	0.9996	0.9858	0.7128	0.7658
Chlorogenic acid	0.9990	0.9926	0.8135	0.8494
Vanillic acid	0.9987	0.9911	0.8296	0.8712
Caffeic acid	0.9999	0.9926	0.8099	0.8442
Syringic acid	0.9999	0.9888	0.8190	0.8560
Epicatechin	0.9997	0.9871	0.7943	0.8250
p-coumaric acid	0.9994	0.9843	0.7561	0.7827
Ferulic acid	0.9994	0.9892	0.7704	0.8043
Sinapic acid	0.9995	0.9855	0.8071	0.8367
Cat gallate	0.9998	0.9931	0.8437	0.8722
Epi gallate	0.9994	0.9951	0.8402	0.8661
Quer-gluc	0.9995	0.9869	0.7494	0.7795
Quercetin	0.9995	0.9901	0.8090	0.8271

**Table A3:** Concentrations (mg/L) and standard deviation beer phenolics as determined by LC-MS/MS for young and aged beer. ND = Not Detected. Means for treatment pairs in bold are statistically different at 95% confidence interval by *t*-test.

Time	Treatment	Gallic acid	Protocate-chuic Acid	p-Hydr-oxybenzoic Acid	Catechin	Vanillic Acid	Caffeic Acid	Epicate-chin	p-Coumaric Acid	Ferulic Acid	Sinapic Acid	Quercetin-Glucoside	Quercetin
Control	Young	**0.325 (0.048)**	**0.329 (0.033)**	0.302 (0.020)	**3.126 (0.266)**	**0.393 (0.047)**	**0.135 (0.01)**	**0.243 (0.061)**	**0.31 (0.005)**	**0.439 (0.024)**	**1.991 (0.141)**	0.422 (0.022)	0.160 (0.163)
	Aged	**0.2 (0.015)**	**0.198 (0.021)**	0.284 (0.026)	**2.558 (0.114)**	**0.309 (0.018)**	**0.076 (0.016)**	**0.235 (0.103)**	**0.209 (0.024)**	**0.383 (0.009)**	**1.55 (0.054)**	0.238 (0.048)	0.024 (0.042)
1H	Young	0.183 (0.062)	0.195 (0.048)	**0.225 (0.046)**	**3.131 (0.279)**	0.333 (0.045)	0.09 (0.035)	**0.21 (0.06)**	0.564 (0.054)	0.424 (0.048)	1.82 (0.251)	**0.33 (0.084)**	0.032 (0.028)
	Aged	0.174 (0.088)	0.147 (0.084)	**0.330 (0.039)**	**2.071 (0.125)**	0.316 (0.061)	0.075 (0.033)	**0.193 (0.041)**	0.304 (0.013)	0.344 (0.022)	1.419 (0.06)	**0.246 (0.022)**	0.071 (0.106)
2H	Young	**0.102 (0.014)**	**0.116 (0.046)**	**0.186 (0.015)**	2.887 (0.512)	0.258 (0.043)	0.062 (0.01)	**0.15 (0.081)**	0.52 (0.078)	**0.379 (0.043)**	1.572 (0.116)	0.232 (0.012)	**0.023 (0.041)**
	Aged	**0.263 (0.000)**	**0.241 (0.001)**	**0.283 (0.026)**	2.141 (0.252)	0.219 (0.002)	0.085 (0.012)	**0.08 (0.08)**	0.255 (0.012)	**0.303 (0.027)**	1.278 (0.068)	0.223 (0.021)	**0.036 (0.036)**
3H	Young	0.297 (0.058)	0.32 (0.061)	0.295 (0.075)	**3.467 (0.229)**	0.430 (0.050)	0.142 (0.025)	0.195 (0.104)	0.637 (0.091)	0.491 (0.048)	2.179 (0.203)	0.421 (0.079)	0.137 (0.075)
	Aged	0.316 (0.006)	0.383 (0.104)	0.470 (0.207)	**2.269 (0.22)**	0.299 (0.096)	0.163 (0.09)	0.111 (0.096)	0.402 (0.164)	0.452 (0.192)	1.715 (0.375)	0.316 (0.103)	0.062 (0.056)
6H	Young	**0.188 (0.031)**	0.198 (0.035)	**0.258 (0.054)**	3.292 (0.2)	0.378 (0.065)	**0.09 (0.003)**	**0.188 (0.051)**	0.542 (0.007)	0.458 (0.052)	1.873 (0.266)	0.310 (0.065)	**0.007 (0.012)**
	Aged	**0.25 (0.004)**	0.255 (0.011)	**0.436 (0.031)**	3.005 (0.117)	0.404 (0.005)	**0.133 (0.008)**	**0.291 (0.047)**	0.398 (0.013)	0.448 (0.047)	1.853 (0.183)	0.420 (0.086)	**0.262 (0.168)**
12H	Young	0.259 (0.037)	0.277 (0.005)	0.264 (0.003)	2.915 (0.047)	0.316 (0.013)	0.111 (0.004)	**0.255 (0.037)**	0.489 (0.01)	0.398 (0.006)	1.602 (0.06)	0.299 (0.048)	**0.057 (0.005)**
	Aged	0.317 (0.028)	0.276 (0.055)	0.328 (0.079)	2.412 (0.38)	0.302 (0.041)	0.128 (0.024)	**0.224 (0.106)**	0.321 (0.039)	0.336 (0.066)	1.46 (0.231)	0.322 (0.055)	**0.099 (0.051)**
24H	Young	0.231 (0.034)	0.226 (0.037)	**0.216 (0.033)**	2.212 (0.321)	0.308 (0.046)	**0.068 (0.01)**	0.159 (0.044)	0.369 (0.046)	0.315 (0.028)	1.295 (0.128)	0.175 (0.020)	**ND**
	Aged	0.284 (0.014)	0.25 (0.051)	**0.323 (0.051)**	2.054 (0.27)	0.317 (0.049)	**0.092 (0.004)**	0.17 (0.06)	0.284 (0.062)	0.302 (0.027)	1.29 (0.138)	0.202 (0.078)	**0.012 (0.021)**
48H	Young	0.249 (0.01)	**0.227 (0.006)**	**0.306 (0.028)**	**3.064 (0.17)**	0.347 (0.027)	**0.09 (0.01)**	**0.203 (0.04)**	0.497 (0.014)	0.449 (0.037)	1.874 (0.176)	0.313 (0.074)	**0.029 (0.05)**
	Aged	0.334 (0.06)	**0.374 (0.01)**	**0.458 (0.068)**	**2.624 (0.149)**	0.377 (0.058)	**0.14 (0.019)**	**0.221 (0.06)**	0.33 (0.009)	0.428 (0.061)	1.797 (0.32)	0.362 (0.073)	**0.075 (0.088)**
72H	Young	**0.206 (0.056)**	0.23 (0.029)	0.289 (0.019)	**3.066 (0.427)**	0.392 (0.061)	0.095 (0.015)	**0.188 (0.033)**	0.487 (0.078)	0.428 (0.051)	1.834 (0.299)	0.266 (0.041)	ND
	Aged	**0.338 (0.027)**	0.296 (0.05)	0.334 (0.081)	**2.057 (0.259)**	0.293 (0.123)	0.121 (0.033)	**0.206 (0.052)**	0.262 (0.041)	0.318 (0.046)	1.351 (0.269)	0.273 (0.054)	ND
96H	Young	0.228 (0.019)	0.25 (0.019)	**0.327 (0.031)**	**3.416 (0.197)**	0.399 (0.029)	0.135 (0.013)	**0.255 (0.055)**	0.542 (0.011)	**0.494 (0.014)**	**2.238 (0.082)**	**0.447 (0.014)**	**0.048 (0.023)**
	Aged	0.24 (0.017)	0.24 (0.043)	**0.417 (0.028)**	**2.453 (0.365)**	0.329 (0.035)	0.112 (0.026)	**0.238 (0.002)**	0.296 (0.071)	**0.424 (0.051)**	**1.801 (0.099)**	**0.322 (0.018)**	**0.03 (0.029)**
